# Electrospun Composites of Polycaprolactone and Porous Silicon Nanoparticles for the Tunable Delivery of Small Therapeutic Molecules

**DOI:** 10.3390/nano8040205

**Published:** 2018-03-29

**Authors:** Steven J. P. McInnes, Thomas J. Macdonald, Ivan P. Parkin, Thomas Nann, Nicolas H. Voelcker

**Affiliations:** 1 Future Industries Institute, University of South Australia, Mawson Lakes 5095, Australia; steven.mcinnes@unisa.edu.au; 2 Department of Chemistry, University College London, London WC1E 6BT, UK; tom.macdonald@ucl.ac.uk (T.J.M.); i.p.parkin@ucl.ac.uk (I.P.P.); 3 MacDiarmid Institute for Advanced Materials and Nanotechnology, School of Chemical and Physical Sciences, Victoria University of Wellington, Wellington 6012, New Zealand; thomas.nann@vuw.ac.nz; 4 Monash Institute of Pharmaceutical Sciences, Monash University, Parkville 3052, Australia; 5 Commonwealth Scientific and Industrial Research Organisation (CSIRO), Clayton 3168, Australia

**Keywords:** porous silicon, drug delivery, electrospinning, poly(ε-caprolactone)

## Abstract

This report describes the use of an electrospun composite of poly(ε-caprolactone) (PCL) fibers and porous silicon (pSi) nanoparticles (NPs) as an effective system for the tunable delivery of camptothecin (CPT), a small therapeutic molecule. Both materials are biodegradable, abundant, low-cost, and most importantly, have no known cytotoxic effects. The composites were treated with and without sodium hydroxide (NaOH) to investigate the wettability of the porous network for drug release and cell viability measurements. CPT release and subsequent cell viability was also investigated. We observed that the cell death rate was not only affected by the addition of our CPT carrier, pSi, but also by increasing the rate of dissolution via treatment with NaOH. This is the first example of loading pSi NPs as a therapeutics nanocarrier into electronspun PCL fibers and this system opens up new possibilities for the delivery of molecular therapeutics.

## 1. Introduction

Synthetic or natural biocompatible polymers are commonly considered as candidates to develop scaffolds for tissue engineering [1]. Effective scaffolds for tissue engineering need to consist of materials that are highly porous, fibrous, biocompatible, biodegradable, and cause no harm to the immune system. It is also important that the complex function of the scaffold mimics the extracellular matrix (ECM), the vital model in providing structural and biochemical support to human cells.

Using compatible biomaterials such as poly(ε-caprolactone) (PCL), chitosan (CS), or gelatin (GEL) is a common approach to engineer a variety of tissue types. The biodegradable polyester PCL is among the most studied scaffolds in tissue engineering and is approved by the United States Food and Drug Administration (FDA, Silver Spring, MD, USA) [2]. PCL has high plasticity, ductility, and ester linkages, allowing for a slow degradation rate from hydrolysis, which are all useful characterization tools for these scaffolds [3]. Biocompatible nanofibers (NFs) produced by electrospinning have been a popular scaffold material due to their similar characteristics to the ECM. PCL NFs have been shown to form suitable interwoven porous scaffolds [4,5,6,7], which assist in the connection of tissues and vessels. These NFs have also been shown to be appropriate structures to mimic the ECM, due to their ability to promote the adhesion and proliferation of cells [8,9]. Additionally, PCL has previously been shown to support a wide range of range of cell types, and its biodegradable features render it an excellent candidate for carrying therapeutic molecules [5].

Porous silicon (pSi) is another example of a biomaterial, and has several unique properties making it attractive for both in vivo and ex vivo applications [10]. pSi is formed by the anodization of crystalline silicon wafers in hydrofluoric acid (HF). pSi nanostructures are easily tailored by altering the wafer resistivity, HF concentration, and current density [11]. Furthermore, pore sizes may be tuned in diameter from a few nanometers to several microns, achieving surface areas of up to 800 m^2^/g [12]. The biocompatibility of pSi has been demonstrated in several animal models [13]. Bimbo et al. showed that the biodistribution of pSi nanoparticles (pSi NPs) does not induce toxicity or inflammatory responses while displaying excellent in vivo stability in rats [14]. Ex vivo applications for pSi include implantable biosensors [15], cancer diagnostics [16], and wound healing [17]. Previously investigated in vitro functionalized pSi NPs and immune cell interactions revealed that even at concentrations of 250 mg/mL, there were no significant cytotoxicity effects [18]. The surface chemistry of pSi is important to load different drugs where a range of in vivo and in vitro studies have demonstrated the biocompatibility of surface-modified pSi [19]. We have reported on composite pSi and PCL membranes implanted into the subconjunctival space of rats [20]. These membranes did not erode or cause inflammatory responses in the tissue surrounding the implant and there was also no evidence of vascularization. Our more recent work investigated the utility of surface-modified pSi membranes as a scaffold for the transfer of oral mucosal cells to the eye. We found that pSi scaffolds supported and retained transplanted rat oral mucosal epithelial cells both in vitro and in vivo [21]. Furthermore, pSi is not limited to biological applications whereby our previous work has also shown its adaptability in solar energy conversion. This verifies pSi as a versatile nanomaterial which can be used in a variety of applications [22].

Electrospinning uses an electrical charge to draw fine fibers from a liquid polymer solution to a charged collector plate. These fibers are typically one-dimensional (1-D) porous structures, which range from submicron to several nanometers in size [23]. Electrospun fibers and in particular NFs offer promising properties such as large surface area to volume ratio, surface flexibility, and superior tensile strength [24,25]. NFs are exciting candidates for a range of applications such as drug delivery [26], biosensing [27], photovoltaic devices [28], and energy storage [29]. An imperative requirement for drug delivery systems is the generation of porous scaffolds to accommodate cells in guiding their growth and regeneration in three dimensions (3-D). This can be achieved using multiple layers of electrospun materials, which produce 3-D fibrillar NF mats [30]. NFs that are used in drug delivery typically follow one of two designs: firstly, for NFs with homogenous structures, the drug or target molecule may be dispersed throughout the fibrous polymer matrix. Secondly, core-shell or coaxial NFs may be fabricated whereby a polymer covers the matrix carrying the drug [31,32]. The drug diffusion mechanism and drug release kinetics are different for the two designs. For example, in homogenous NFs, the drug must travel progressively long distances in order to diffuse throughout the outer edge of the NFs, meaning that the rate of release typically decreases with time. In contrast, core-shell NF systems enable stable diffusion rates of the drugs due to the structure of the system; however, their preparation is much more complex. A core advantage of using NFs is their structure (diameter, density, and thickness), which can be easily tailored by varying the process parameters or combining other nanomaterials within the fibrous structures [33,34]. Furthermore, fiber diameter is tunable and dependent on polymer molecular weight, sol-gel concentration, flow rate, applied voltage, needle tip size, and hydrolytic degradation of NFs [35].

Herein, we combine the favorable properties of both pSi and PCL to manufacture drug eluting composite electrospun PCL fibers containing pSi NPs. We investigate the release kinetics of the small molecular drug camptothecin (CPT). In an effort to control the release kinetics, we investigate the difference of release properties of materials loaded directly with CPT and those with the equivalent CPT amount preloaded into pSi NPs. We also investigate the effect of pretreating the PCL fibers with sodium hydroxide (NaOH) to enhance their wettability and degradation rate [36].

## 2. Materials and Methods

### 2.1. Chemicals

Hydrofluoric acid (HF) 48% (Merck), dichloromethane (CH_2_Cl_2_, Labserv, analytical grade, 99.5%), and polycarolactone (80,000 average m wt) were purchased from Sigma-Aldrich and used as received. Methanol (Merck, analytical grade, 99.5%), DCM (Chemsupply, analytical grade, 99.5%), and ethanol (Ajax, absolute, 100%) were used without further purification. *N*,*N*-dimethylformamide (DMF, EMD Chemicals, Overijse, Belgium) was purified via standard laboratory protocols including drying over MgSO_4_ followed by distillation at reduced pressure [36]. Milli-Q water was obtained from an Advantage A10 water purification system provided by Merck Millipore (water resistivity of 18.2 MΩcm at 25 °C, Total Organic Carbon (TOC) < 5 ppb). Dulbecco’s phosphate buffered saline (PBS) solution and fluorescein isothiocyanate (97.5%, FITC) were purchased from Sigma Aldrich and used as received.

### 2.2. Fabrication of pSi NPs

pSi NPs were fabricated from p-type Si wafers (boron-doped, resistivity 0.0008–0.0012 Ω cm, <100>) supplied by Siltronix (Archamps, France). The wafer was anodized in an 18-cm^2^ etching cell in 3:1 HF:ethanol (*v*/*v*) solution with a square wave form comprising an initial current density of 50 mA/cm^2^ for 7.3 s and a second current density of 400 50 mA/cm^2^ for 0.4 s. This two-step cycle was repeated continuously for 1 h, generating a pSi film with alternating low and high porosity layers. The etched layer was removed from the Si substrate via electropolishing in 1:20 HF:EtOH at 4 mA/cm^2^ for 4 min and 10 s. Subsequently, the pSi membrane was sonicated for 16 h in DMSO to generate chemically oxidized pSi NPs. These nanoparticles were sized by passing through a 220-nm polytetrafluoroethylene (PTFE) syringe filter, followed by the collection of the pellet after centrifugation at 22,000× *g*. This filtration and centrifugation allowed for the removal of large and small nanoparticles and facilitated the harvest of reasonably uniformly sized NPs that permanently remained in solution.

### 2.3. CPT Loading of pSi NPs and PCL Composite Materials

pSi NPs were placed into an Eppendorf tube, to which a known volume (50–200 µL) of CPT solution of approximately 4.6 mg/mL CPT in dry distilled DMF was added. This mixture was allowed to incubate for 2 h before the particles were dried under vacuum (10 mm Hg) in a desiccator. The total loadings were calculated based on the mass of pSi placed into the tube originally; for example, a typical loading used 200 µL of 4.6 mg/mL CPT and 15.0 mg of pSi, resulting in a loading of 61.3 µg of CPT per mg of pSi. Each individual batch loading was used to convert the release amounts into percentages.

To load the PCL NFs, a predetermined mass of pSi was added to the spinning solution based on the loading of the pSi NPs. The final loading of CPT in the spinning solution was normalized to contain 0.63 mg of CPT for both the PCL + CPT materials and the PCL + pSi-CPT materials. In the above example, 10.3 mg of CPT loaded pSi NPs would be added to the spinning solution. To ensure a homogeneous distribution of the pSi NPs in the spinning solution, the particles were briefly sonicated in a small amount of acetone before injection into the spinning solution. After injection, the solutions were stirred as best as possible and then placed into the syringe for spinning.

### 2.4. Water Contact Angle (WCA) Measurements

The WCA was measured by placing a 1-µL drop of water on the sample surface and capturing a digital image using a Panasonic Super Dynamic wv-BP550 Closed Circuit TV camera. The contact angle measurements were analyzed by Scion Image for Windows Framegrabber software (Beta version 4.0.2).

### 2.5. Electrospinning of PCL and NaOH Treatments

In a typical synthesis, a 5-mL solution of 10% PCL in acetone with pSi NPs was electrospun from a 23 G stainless steel needle. The mass of pSi added to the electrospinning solution was dependent on the loading of the particular batch of pSi; however, for all of this work the mass of pSi used was balanced to facilitate a loading equivalent to 0.63 mg of CPT in the pSi + CPT composite material. Hence, every material spun and tested for drug release contained the same initial loading of CPT. The needle was then connected to a high-voltage supply (Gamma High Voltage Research, Ormand Beach, FL, USA). The solution was fed at a rate of 0.5 mL/h using a syringe pump (PHD 2000, Harvard Apparatus, Holliston, MA, USA). A piece of flat aluminum foil was placed 10 cm below the tip of the needle as a collector plate. The voltage for the electrospinning was set to 15 kV and was conducted in a controlled temperature environment (25 °C).

### 2.6. Scanning Electron Microscopy (SEM)

SEM was performed on an FEI Quanta 450 FEG environmental SEM fitted with a Secondary Electron Detector (SED) detector and operated at 30 keV with a spot number of 2. To help facilitate the dissipation of charge build-up, samples were coated with a 5-nm thick layer of Pt prior to analysis, according to our standard laboratory protocol.

### 2.7. X-ray Photoelectron Spectroscopy (XPS)

XPS measurements were recorded on a Thermo Scientific K-alpha spectrometer with monochromatic Al-K_α_ radiation at University College London. This involved obtaining a monatomic depth profile of the NFs using an ion beam to etch layers of the surface revealing subsurface information. The etching was performed for 200 s, which was calibrated to penetrate ~50 nm into the surface. Peak positions were calibrated to carbon (285 eV) and plotted using the CasaXPS and qtiplot software.

### 2.8. Energy-Dispersive X-ray Spectroscopy (EDX)

EDX was obtained using an Oxford Instruments UTW Energy Dispersive Spectroscopy (EDS) detector running ISIS software. The EDS detector was ran through a JEOL JSM-6301F Field Emission SEM.

### 2.9. Fluorescence Microscopy

Fluorescence microscopy was performed on an Eclipse 50*i* microscope equipped with a D-FL universal epi-fluorescence attachment and a 100-W mercury lamp (Nikon Instruments, Tokyo, Japan). Fluorescence images were captured with a Charged-Coupled Device (CCD) camera (Nikon Instruments, Tokyo Japan), using the following fluorescence filters. Blue channel (violet excitation, blue emission): excitation: 385–400 nm (bandpass, 393 CWL), dichromatic mirror: 435–470 nm (bandpass), and barrier filter wavelength: 450–465 nm (bandpass, 458 CWL). Green channel (blue excitation, green emission): excitation: 475–490 nm (bandpass, 483 CWL), dichromatic mirror: 500–540 nm (bandpass), and barrier filter wavelength: 505–535 nm (bandpass, 520 CWL). Red channel (green excitation, orange/red emission): excitation: 545–565 nm (bandpass, 555 CWL), dichromatic mirror: 570–645 nm (bandpass), and barrier filter wavelength: 580–620 nm (bandpass, 600 CWL). Images were analyzed using NIS-elements v3.07 software (Nikon Instruments, Tokyo, Japan).

### 2.10. Drug Release

CPT release was monitored via fluorimetry and performed on a Perkin Elmer Instruments LS55 luminescence spectrometer with an excitation wavelength of 340 nm and emission wavelength of 434 nm. The slit width was set to 3 nm and the photomultiplier voltage was set to 775 V. The cumulative release data of CPT into 3 mL of PBS was monitored over a 17-h period. Release rates were calculated from the slope of release curves obtained. The actual amount of CPT released was calculated with reference to a calibration curve and normalized to the surface area of the sample to give the amount of CPT released per cm^2^. This allowed the CPT release data to be directly compared between each of the samples. A minimum of three release curves was averaged to produce the release curves. The release of CPT was performed in PBS at pH 7.4 and pH 1.8. Despite the low solubility of CPT in aqueous solutions (14.2 ± 2.9 μM) [37], sink conditions were maintained for all release experiments (maximum release concentrations were below 1.4 μM).

### 2.11. Cytotoxicity Assay

SH-SY5Y cells (human neuroblastoma cells), were cultured in Dulbecco’s Modified Eagle’s Medium (DMEM) supplemented with 10% FBS, 100 U/mL penicillin, and 100 µg/mL streptomycin (Invitrogen) as previously described. SH-SY5Y cells were placed in wells of a 96-well plate at 15,000 cells per well. After one day, the cultured cells were incubated with the prepared CPT-loaded and CPT-free PCL NFs (the following set of samples was used: PCl only, PCl + CPT, PCl + pSi-CPT). PCl NFs in DMEM (10% FBS, 100 U/mL penicillin, and 100 µg/mL streptomycin) were incubated for 24, 48, and 96 h at 37 °C and 5% CO_2_ prior to cell viability measurements. Controls were generated by incubating SH-SY5Y cells in DMEM without a PCl NF for an identical incubation period.

To determine the effect of the microparticles treatment on cell viability, the percentage of live and dead cells, lactate dehydrogenase (LDH) released in culture supernatants was measured using an established assay (Abcam LDH-Cytotoxicity Assay Kit II) according to the manufacturer’s instructions. After 24, 48, and 96 h of incubation with microparticles, 100 µL of the cell suspension was removed and centrifuged at 600× *g* for 10 min, and the supernatant was transferred to a 96-well plate. To each well, 100 μL LDH reaction mix (Abchem) was added. After 30 min of incubation at room temperature, the absorbance at 450 nm was measured. All experiments were repeated at least three times.

## 3. Results and Discussion

### 3.1. Material Characterization

The characterization of pSi NPs was performed in our earlier work [38]. Briefly, the particle size was found to be in the range of 161 ± 58 nm and the particles possessed a pore size of 33 ± 7 nm. The characterization of the pSi NPs can be found in the Supporting Information (ESI, Figure S1).

**Figure 1:**
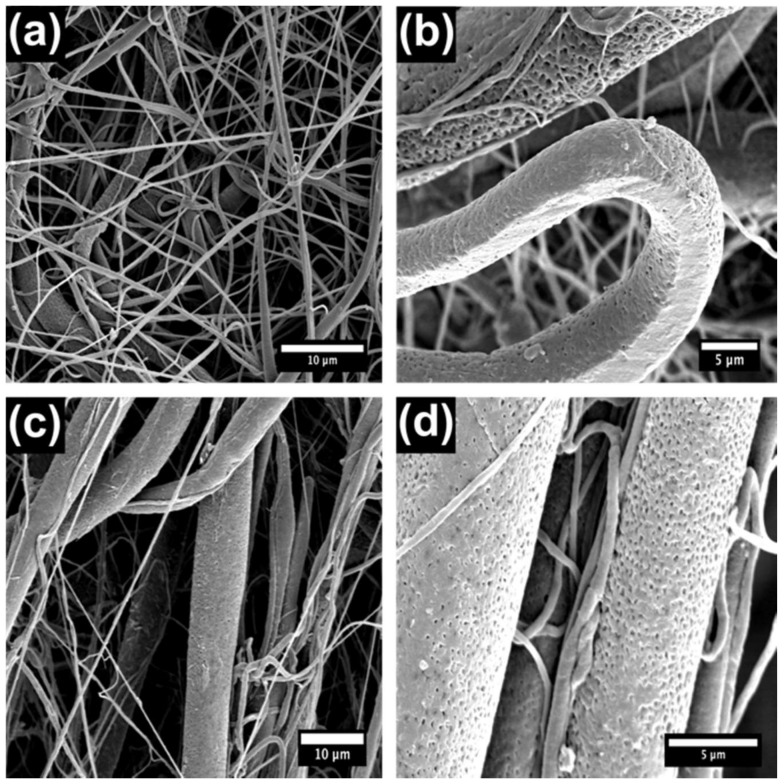
SEM characterization of poly(ε-caprolactone) nanofibers (PCL NFs) with and without treatment with NaOH. (**a**,**b**) No NaOH treatment and (**c**,**d**) 24-h NaOH treatment.

**Figure 2:**
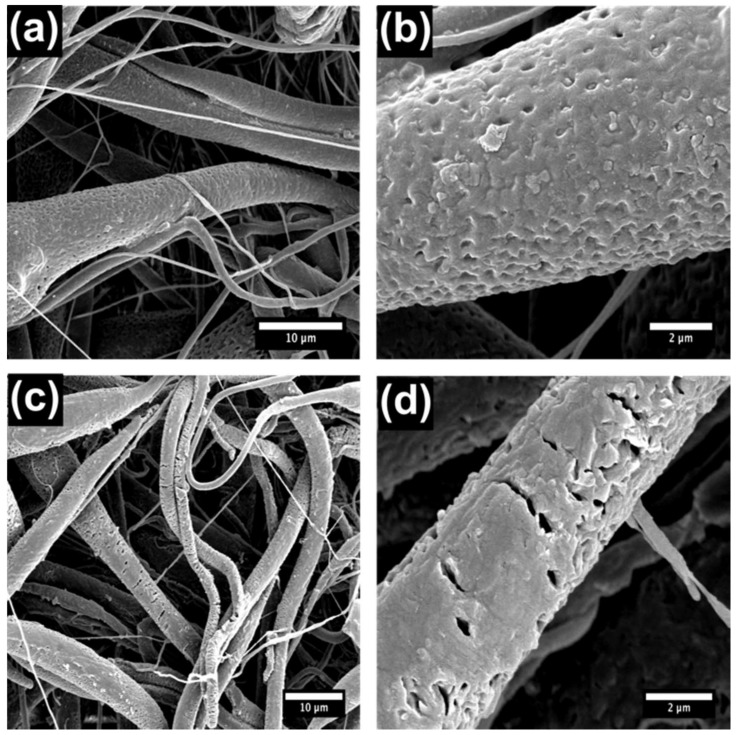
SEM characterization of PCL + porous silicon (pSi) NFs with and without treatment with NaOH. (**a**,**b**) No NaOH treatment; (**c**,**d**) 24-h NaOH treatment.

Electrospun materials were removed from the Al foil backing and folded until they were eight layers thick. Subsequently, 3-mm diameter NF discs were punched from the PCL sheet using a hole punch. The NFs were of homogeneous size and thickness and on average weighed 1.43 ± 0.66 mg. NaOH is known to speed up the hydrolysis of the PCL polymer backbone [36]. We calculated an average % mass loss of 3.5% PCL per hour of exposure to NaOH. We anticipated the base treatment to enhance the speed at which the PCL degraded and hence control the release rate of the CPT. Scanning electron microscopy (SEM) of the NFs is shown below (Figure 1 and Figure 2) and indicates that the PCL NF diameter ranges between several hundred nanometers and a few microns, which is consistent with previous reports [39,40]. However, from the SEM characterization in Figure 1, it is clear that the use of NaOH on the prepared fibrous networks destroyed the smaller fibers (<500 nm) in the network, leaving behind larger fibers which in fact slowed the release of CPT rather than speeding it up.

**Figure 3:**
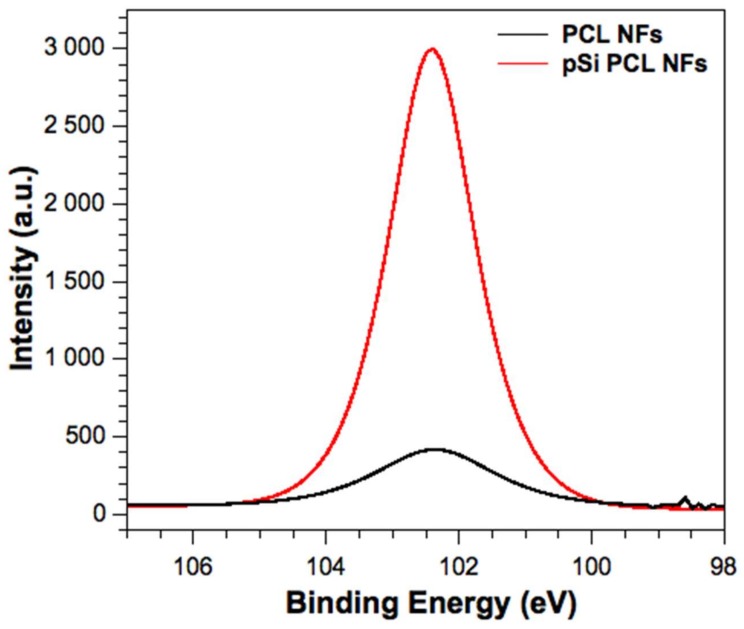
High-resolution Si 2p X-ray photoelectron spectroscopy (XPS) characterization of PCL and PCL + pSi NFs.

X-ray photoelectron spectroscopy (XPS) of the PCL and PCL with pSi (PCL + pSi) NFs was performed to determine the presence of pSi in the PCL fibers; the resulting XPS of the Si 2p spectra is shown below in Figure 3. Given the small amounts of silicon expected to be preset, and its likelihood of appearing on the XPS spectra as a surface contaminant, XPS for PCL NFs and pSi PCL NFs was obtained by measuring a depth profile of each of the samples. XPS depth profiling involved using an ion beam to etch off subsequent layers, revealing subsurface information. The depth profile corresponded to a penetration of roughly 50 nm for each NF sample. The more intense Si 2p signal in the pSi PCL NFs suggests that the pSi NPs are well embedded into the surface of the NFs. Figure 3 shows characteristic Si 2p peaks at 103 eV for both pSi PCL and PCL NF samples. This binding energy corresponds to oxidized pSi (pSiO_2_), which is expected after the pSi NPs are sonicated in DMSO and subject to oxidation (see experimental section). The small Si 2p signal present in the PCL NF sample is a likely a contaminant, since siloxane compounds are widely used as lubricants and release agents in polymer materials. Siloxanes have a strong tendency to migrate towards polymer surfaces and at small bulk concentrations, resulting in segregation to the outer surface and causing high surface contamination [41]. The contaminant is typically characterized as a broad, low intense siloxane peak, which is consistent with the high resolution Si 2p spectrum shown in Figure 3 [41]. Furthermore, given that the depth profile measurements only showed an increase in Si 2p signal for the pSi PCL NF sample, this suggests that the pSi NPs are well within the pSi PCL NF network. The binding energies with their chemical assignments are shown in the Electronic Supporting Information (ESI), Table S1. EDX analysis before and after NaOH treatment also corroborated the XPS findings (ESI, Figure S2).

**Figure 4:**
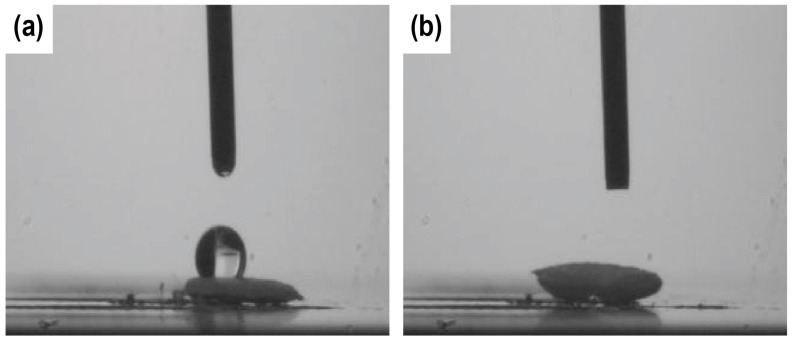
Water contact angle (WCA) measurements of (**a**) PCL without NaOH treatment and (**b**) PCL with 1-h NaOH treatment.

The static water contact angle (WCA) of the PCL fibers was 113.5 ± 8.4° (Figure 4a). However, after a 1-h treatment of the PCL with NaOH, the PCL NFs completely absorbed the 1-µL droplet placed onto the surface (Figure 3b). This complete wetting of the water droplet into the PCL NFs could be due to both the increase of the hydrophilicity of the fibers [36] and the opening up of the porous mesh due to the breakage of smaller NFs and the pitting of the thicker NFs after the NaOH treatment (Figure 4).

### 3.2. Drug Loading and Release

Throughout this work, the mass of pSi added to the electrospinning solution was balanced to facilitate the loading of CPT, according to our methodology as detailed in the experimental section. Every material spun and tested for drug release contained the same initial loading of CPT.

**Figure 5:**
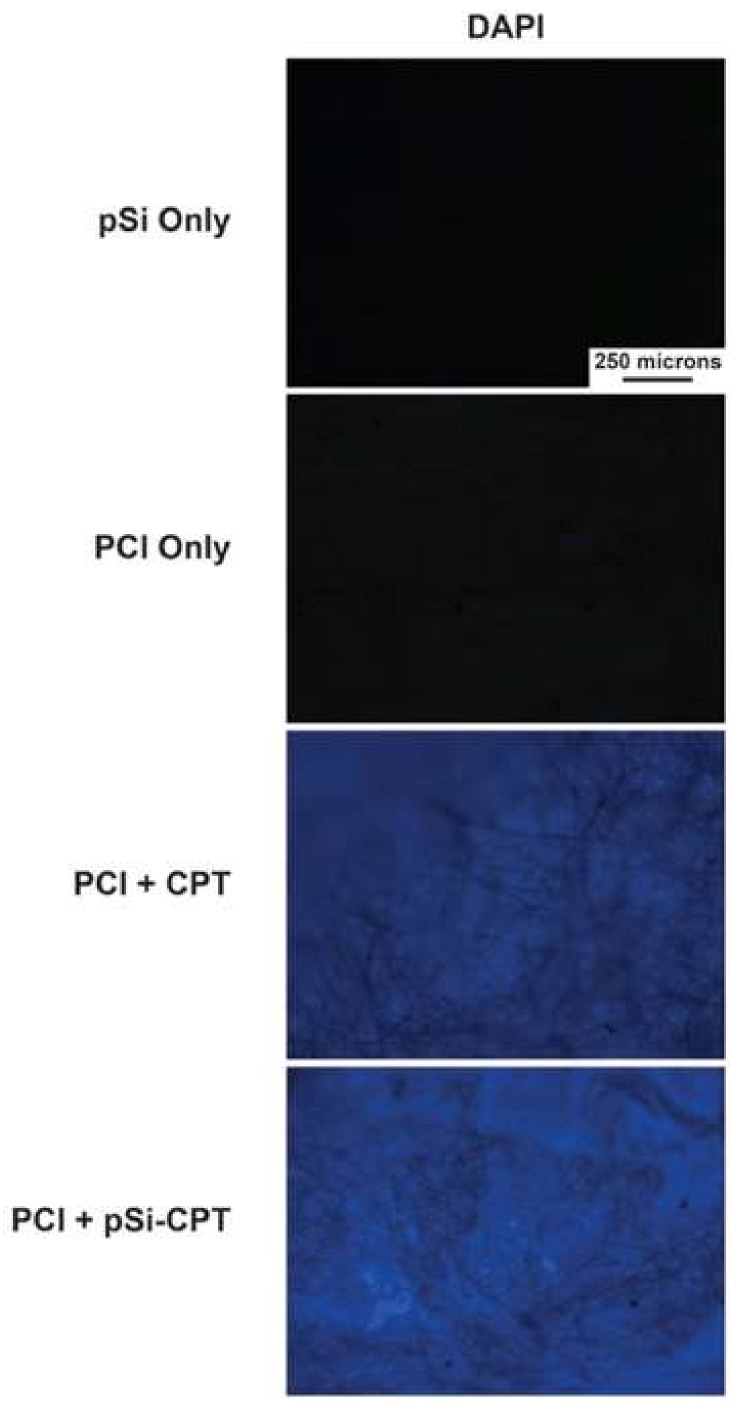
Fluorescence microscopy images of the original pSi NPs, PCL materials, and the two composite materials in the DAPI channels. All images are taken at the same exposure settings to facilitate qualitative comparison.

Fluorescence microscopy of the loaded and unloaded materials (Figure 5) below was conducted in an attempt to track and visualize the location of the pSi NPs and CPT throughout the PCL + pSi composite materials. Figure 5 demonstrates the fluorescence of the pSi NPs, the PCL only, and the two different composite preparations in the DAPI (CPT-sensitive) channel. It is clear that the pSi NPs and the PCL themselves showed no auto-fluorescence. However, upon the addition of CPT, there was a notable increase in the fluorescence in the DAPI channel for both CPT only and pSi-CPT containing samples, indicating that CPT was located throughout the network of both sample preparations.

**Figure 6:**
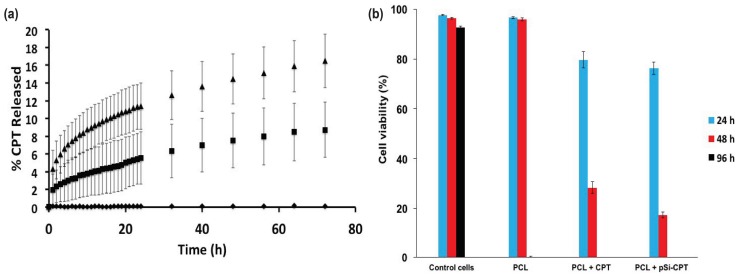
(**a**) Release curves of camptothecin (CPT) from PCL + CPT and PCL + pSi-CPT composite materials without NaOH treatment. ♦ = PCL control material, ■ = PCl + CPT, ▲ = PCl + pSi-CPT; (**b**) Viability of SH-SY5Y human neuroblastoma cells upon incubation with PCL and PCL + pSi-composite materials with or without CPT loading (*n* ≥ 3).

**Figure 7:**
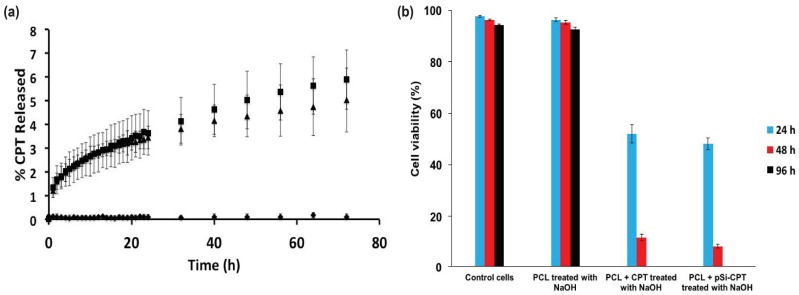
(a) Release curves of CPT from (**a**) PCL + CPT composites and (**b**) PCL + pSi-CPT composite materials after 1-h NaOH treatment. ♦ = PCL control material, ■ = PCL + CPT, ▲ = PCL + pSi-CPT. Viability of SH-SY5Y human neuroblastoma cells upon incubation with PCL and PCL + pSi-composite materials with or without CPT loading and NaOH treatment (*n* ≥ 3).

CPT drug release was monitored from the PCL + CPT and PCL + pSi-CPT composite materials both with and without the NaOH treatment (Figure 6a and Figure 7a). The PCL only control material did not show any release or interference in the CPT signal measured at 434 nm. Before NaOH treatment, the PCL + pSi-CPT showed significantly higher % release from the pSi-containing composite compared to the PCL + CPT (Figure 6a), possibly due to the pSi creating weak points in the surface of the PCL fibers, which are more susceptible to hydrolysis and aid in speeding the dissolution of the PCL fiber.

The untreated PCL + CPT composite and the 1-h NaOH treated PCL + CPT composite showed release rates of 8.72 ± 3.13% and 5.90 ± 1.25%, respectively (Figure 6a and Figure 7a). The PCL only materials (Figure 6a and Figure 7a) did not display any release in either the treated or untreated case. The same materials also possessed average release rates of 0.121%/h and 0.082%/h, respectively. The untreated PCL + pSi-CPT composite and the 1-h treated PCL + pSi-CPT composite materials showed final release percentages of 16.48 ± 3.04% and 5.03 ± 1.35%, respectively (Figure 6a and Figure 7a). The same materials also possessed average release rates of 0.229%/h and 0.070%/h, respectively. 

Modeling of the release data (Table 1) revealed that all of the materials best fit the Higuchi model. This indicates that the release followed a mostly diffusion-controlled process out of the PCL fiber networks. Using the Ritger-Peppas model to assess the release mechanism via the ‘*n*’ exponent revealed that the ‘*n*’ exponent was always between 0.45 and 1. Therefore, we can assume that the release mechanism is a non-Fickian (anomalous) diffusion for all samples. This indicates a mixture of release from the CPT diffusing out of the fibers and some additional effects from the PCL polymer molecules as the water infiltrates the polymer network.

### 3.3. Cell Viability Assay

Figure 6b and Figure 7b show the cell viability in an LDH assay for all PCL composite samples over a 4-day time course. The control (untreated) neuroblastoma cells and the cells treated with PCL only and PCL only with NaOH treatment (1 h) showed virtually 100% cell viability over the 4-day time period (Figure 6b and Figure 7b). This confirms that neither the pSi nor any residual NaOH left behind after treatment caused any cytotoxic effect (at least for the LDH assay). In stark contrast, the CPT-loaded PCL and PCL + pSi materials (Figure 6b) showed a rapid onset of decreased viability within the first day and this only increased over the full 4-day time course. The viability of the cells exposed to PCL + CPT and PCL + pSi-CPT samples not pretreated with NaOH showed decreased viability from 80% to 28% and 76% to 17%, respectively, from day 1 to 2 (Figure 6b). Both samples had 0% cell viability at day 4. Similarly, the same two sample sets pretreated for 1 h in NaOH dropped in viability from 52% to 12% and 48% to 8%, respectively, from day 1 to 2 (Figure 7b). Again, both samples had 0% cell viability at day 4 (Figure 7b). These biological results demonstrate that the cell death rate was not necessarily affected by the addition of the pSi as a carrier for the CPT, but it was affected by increasing the dissolution rate via treatment with NaOH.

### 3.4. General Discussion

PCL materials were successfully fabricated via electrospinning and were able to encase the model drug CPT or pSi NPs preloaded with CPT. To the best of our knowledge, this is the first time pSi NPs have been spun through PCL fibers. This simple entrapment of small molecular drugs in a carrier such as pSi could lead to the development of electrospun constituents that are able to contain sensitive payloads that would normally not be able to be incorporated directly into these materials. NFs with small diameters have a large surface area per unit mass, and this work is just one example of how particles and biological structures can be isolated and protected inside NFs, while remaining accessible for use when needed. Herein, we show that NFs loaded with pSi can be used as convenient carriers for the tunable delivery of small therapeutic molecules [42].

The release rates of the small molecular model drug, CPT, can also be modulated both via incorporation into pSi particles or by direct and intentional degradation (incubation with NaOH) of the PCL scaffold. The ability to affect the release rate of CPT from these materials also gives rise to a significant difference in the cell cytotoxicity of the released CPT on SH-SY5Y cells. This can be exploited for the tunable delivery of therapeutic payloads to areas such as non-resectable tumors. Furthermore, this work opens up the possibility of delivering a range of therapeutic molecules from electrospun fibers, potentially leading to the development of smart scaffolds that can allow for cell growth and infiltration whilst facilitating the release of cell growth-stimulating biological factors.

## 4. Conclusions

PCL polymeric fibers could be combined both directly with a small molecular drug payload and a particulate carrier system for the drug payload. Tunable drug release and subsequent cell death could be achieved by tuning the composition of the PCL composites or pretreating them with NaOH to generate a porosity that enhances dissolution. Our methodology may open the possibility of protecting small therapeutic molecules within a porous carrier, such as porous silicon, during the high voltage spinning process for later sustained long-term release applications.

## Figures and Tables

**Table 1:** Modeling of the release kinetics and mechanism.

Material	Zero-Order	First Order	Higuchi	Hixson-Crowell	Ritger-Peppas	‘*n*’
PCL + CPT	0.899	0.925	0.992	0.558	0.981	0.74
PCL + CPT 1-h NaOH	0.890	0.909	0.991	0.559	0.987	0.70
PCL + pSi-CPT	0.806	0.886	0.966	0.425	0.999	0.63
PCL + pSi-CPT 1-h NaOH	0.823	0.843	0.974	0.531	0.998	0.67

## Supplementary Materials

The following are available online at http://www.mdpi.com/2079-4991/8/4/205/s1, Figure S1: Representative pSi NP TEM microscopy images. Particles were found to be typically 161 ± 58 nm with a pore size of 33 ± 7 nm. Table S1. XPS of PCL and PCL + pSi composite discs. Figure S2: (Top) EDX spectra for PCl fibers after NaOH treatment. (Bottom) EDX spectra for PCL fibers containing pSi NPs after NaOH treatment.
