# Supplementary material for: Electrospun Composites of Polycaprolactone and Porous Silicon Nanoparticles for the Tunable Delivery of Small Therapeutic Molecules
Source: Nanomaterials (Basel). 2018 Mar 29;8(4):205. doi: 10.3390/nano8040205 (PMC5923535; doi:10.3390/nano8040205)
Supplement: Supplementary file 1 [file nanomaterials-08-00205-s001.pdf]

Electronic Supporting Information

Electrospun Composites of polycaprolactone and pSi NP's for the tunable delivery of small therapeutic molecules

Steven J. P. McInnes [1], Thomas J. Macdonald [2], Ivan P. Parkin [2], Thomas Nann [3] and Nicolas H. Voelcker [4,5]*

1. Future Industries Institute, University of South Australia, Mawson Lakes, Australia; steven.mcinnes@unisa.edu.au
2. Department of Chemistry, University College London, London, United Kingdom; tom.macdonald@ucl.ac.uk, i.p.parkin@ucl.ac.uk
3. MacDiarmid Institute for Advanced Materials and Nanotechnology, School of Chemical and Physical Sciences, Victoria University of Wellington, Wellington, New Zealand; thomas.nann@vuw.ac.nz
4. Monash Institute of Pharmaceutical Sciences, Monash University, Parkville, Australia
nicolas.voelcker@monash.edu.au
5. Commonwealth Scientific and Industrial Research Organisation (CSIRO), Clayton, Australia

pSi NP Characterisation

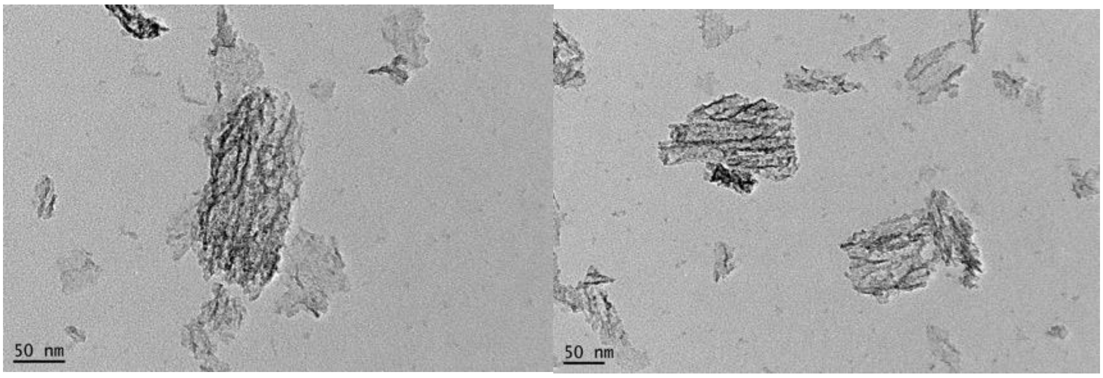

Figure S1: Representative pSi NP TEM microscopy images. Particles were found to be typically 161 +/- 58 nm and a pore size of 33 +/- 7 nm.

Dynamic light scattering (DLS) gave a nanoparticle size of 149 +/- 25 nm.[1]

XPS

Table S1. XPS of PCL and PCL + pSi composite discs.

PCL Nanofibers			pSi PCL Nanofibers		
Name	Binding Energy (eV)	Assignment	Name	Binding Energy (eV)	Assignment
C1s	284.5	C-H	C1s	284.5	C-H
C1s	288.7	C=O	C1s	289.1	C=O
O1s	532.2	C-O	O1s	532.3	C-O
O1s	533.5	C-O	O1s	533.6	C-O
Si2p	102.4	C-Si	Si2p	102.4	C-Si

EDX

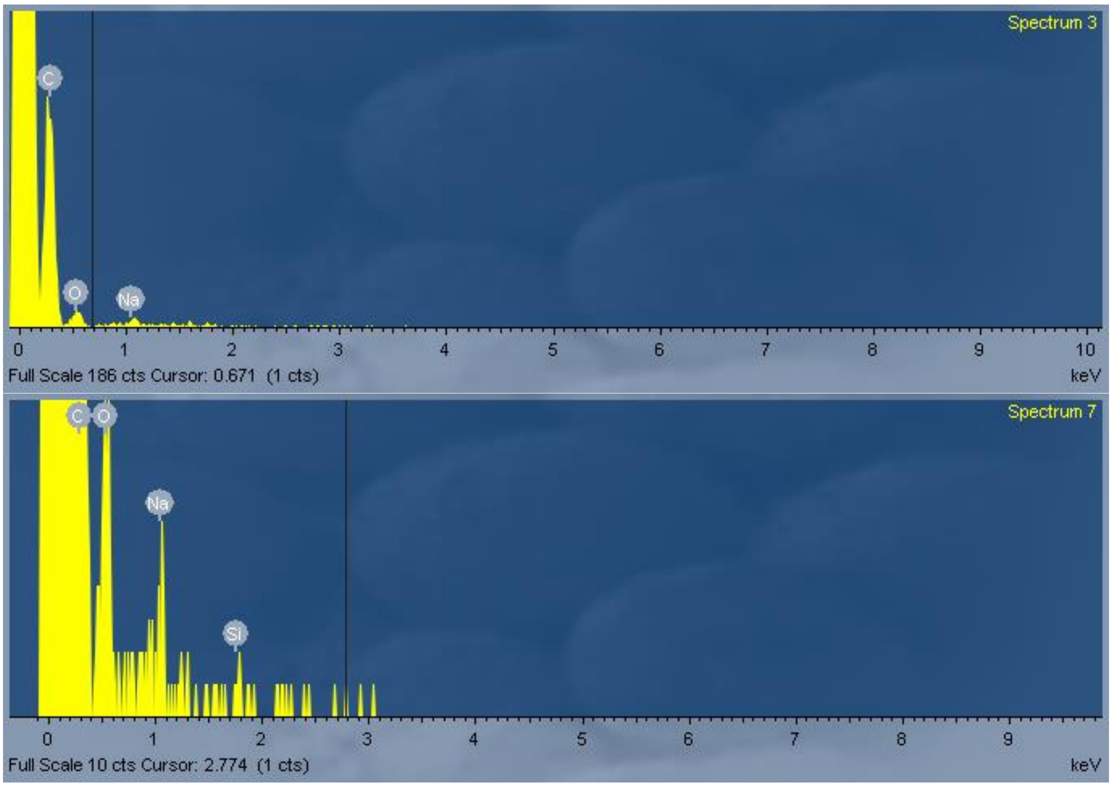

Figure S2: (top) EDX spectra for PCl fibers after NaOH treatment. (bottom) EDX spectra for PCL fibers containing pSi NPs after NaOH treatment.

References:

1) Christopher T. Turner, Steven J. P. McInnes, Elizabeth Melville, Allison J. Cowin and Nicolas H. Voelcker, Delivery of Flightless I Neutralizing Antibody from Porous Silicon Nanoparticles Improves Wound Healing in Diabetic Mice. Adv. Healthcare Mater. 2016**, 6**(2).
